# Supplementary material for: Attractiveness and retention factors for employed physiotherapists in France: A multicentre survey
Source: PLoS One. 2025 Mar 28;20(3):e0320919. doi: 10.1371/journal.pone.0320919 (PMC11952219; doi:10.1371/journal.pone.0320919)
Supplement: Attachment — Submitted filename: PointByPoint.docx [file pone.0320919.s003.docx]

**Point-by-Point Response to Reviewers**

Manuscript ID: PONE-D-24-30478R1
Title: *Attractiveness and Retention Factors for Employed Physiotherapists in France: A Multicentre Survey*

Dear Editor and Reviewers,

We would like to express our gratitude to the reviewers and the editor for their comments, which have helped us improve the manuscript. Below, we provide detailed responses to each point raised. All modifications have been incorporated into the revised manuscript, with track changes.

**Reviewer #1**

**Comment:** All comments have been addressed.

**Response:** We appreciate the reviewer's positive feedback and are pleased that the revisions have addressed their concerns.

**Reviewer #2**

**General Comment**

*"I would like to commend the authors on their consideration of the previous comments and their efforts in reviewing the manuscript. It is much improved - particularly the introduction."*

**Response:** Thank you for your positive feedback. We appreciate your comments and have carefully addressed the remaining minor issues to further improve the clarity and coherence of the manuscript.

**Comment 1: Persistence of "employed physiotherapy" in some sections**

*"The term 'employed physiotherapy' still persists in some text - e.g., in discussion and title Table IV."*

**Response:** We have carefully reviewed the manuscript and replaced all instances of *"employed physiotherapy"* with a more precise and appropriate term. The title of Table IV has also been updated accordingly.

- Line 215: “The results provide a nuanced understanding of the factors influencing the attractiveness of physiotherapy positions in public hospitals.”
- Line 231: “The study revealed several barriers that diminish the appeal of physiotherapy positions in public hospitals.”
- Line 266: “Table VI: 10 measures to improve the appeal of physiotherapy positions in public hospitals.”

**Comment 2: Clarification of Chi-square analysis in Table III**

*"Table III presents results of Chi-square analysis - yet, the categories used for this analysis are not clear. The text mentions 'participants <40 years' - were <40 and >40 years categories used for age in this analysis? Presenting the p-value next to the variable doesn’t communicate the analysis and which category was significantly different."*

**Response:** We have clarified the age categories used in the Chi-square analysis by explicitly stating that the groups were *"<40 years"* and *">=40 years"*. Additionally, we have added the corresponding Chi-square values (χ²) in Table III to provide a clearer representation of the statistical analysis. To improve readability, we have also removed the "current profession" (manager) category from the table, as the results were not significant.

- Line 193: “Conversely, no significant associations were observed between others measured variables”
- Line 196:

Table V : Effect of age on socio-professional outcomes (n=448). For statistical analysis, age was dichotomized into two categories: <40 years and ≥40 years. The chi-square test was used to assess differences between these groups regarding the stated variables.

| **The quality of worklife is important in the choice of your professional activity** | | | | | | |
| --- | --- | --- | --- | --- | --- | --- |
| Age group  n (%) | *Strongly agree* | *Agree* | *Neither disagree nor agree* | *Disagree* | *Strongly disagree* | *Chi-Squared Tests* |
| <40 | 221 (89.5%) | 25 (10.1%) | 1 (0.4%) | 0 (0.0%) | 0 (0.0%) | Χ² = 27.4  p < .001 |
| ≥40 | 141 (70.1%) | 56 (27.9%) | 2 (1.0%) | 2 (1.0%) | 0 (0.0%) |  |
| **Taking personal quality of life into account by management promotes attractiveness** | | | | | | |
| <40 | 177 (71.7%) | 62 (25.1%) | 8 (3.2%) | 0 (0.0%) | 0 (0.0%) | Χ² = 17.2  p = 1.7×10^-3^ |
| ≥40 | 109 (54.2%) | 76 (37.8%) | 13 (6.5%) | 2 (1.0%) | 1 (0.5%) |  |

**Comment 3: Correction of Line 126**

*"Line 126 'This four...' needs correcting."*

**Response:** This typographical error has been corrected in the revised manuscript.

- Line 104: “These four domains were chosen”

**Comment 4: Placement of the "Limitations" section**

*"Discussion - I suggest moving the 'limitations' section to before the conclusions rather than before the interpretation of findings."*

**Response:** We have followed the reviewer's suggestion and moved the "Limitations" section to appear before the conclusions: line 168.

**Comment 5: Clarification on financial revaluations and salary revisions (Lines 272-276)**

*"Lines 272-276 - This section states that financial revaluations and attractiveness bonuses have had limited success. Yet, revising salaries is the first measure the authors suggest. Are the authors suggesting profession-wide policy change is needed here to align all public institutions - if so, this needs to be clearer."*

**Response:** We acknowledge the need for clarification and have revised this section to explicitly state that salary adjustments should be considered as part of a broader structural reform aimed at ensuring consistency across public institutions. The revised text now highlights that while previous financial incentives had limited success, a systematic and profession-wide policy change could enhance long-term attractiveness by addressing disparities in remuneration and career progression.

- Line 243: “While financial revaluations and attractiveness bonuses have had limited success when implemented in isolation, they remain essential as part of a broader, systemic reform. A structured salary revision, combined with institutional policies recognizing additional qualifications and improving working conditions, could help address the deeper structural challenges of the profession.”

**Additional Journal Requirements**

We have also reviewed and updated the reference list to ensure completeness and accuracy. No retracted papers have been cited.

We hope that these revisions adequately address all concerns. Please let us know if further modifications are needed.

Kind regards,
