# Supplementary material for: Attractiveness and retention factors for employed physiotherapists in France: A multicentre survey
Source: PLoS One. 2025 Mar 28;20(3):e0320919. doi: 10.1371/journal.pone.0320919 (PMC11952219; doi:10.1371/journal.pone.0320919)
Supplement: Attachment — Submitted filename: Review plos one.docx [file pone.0320919.s002.docx]

**Point by Point response**

We thank the reviewers for their valuable comments and suggestions, which have significantly improved the manuscript. Below is a point-by-point response to the reviewers' comments.

The manuscript has been extensively revised to address the reviewers' feedback. Key improvements include enhanced clarity in language, restructuring of sections for better narrative flow, and the addition of details to contextualize findings. Specific changes are outlined below for each reviewer comment.

# 1. Reviewer #1

## 1.1. General Comments:

- ***"Thanks you for the opportunity to review this manuscript. The subject is topical and this study has the potential to be of interest to those interest in health workforce development. Whilst the methods were appropriate, and much of the conduct and reporting of the study are commendable, there is weakness in the writing and the contextualization of findings.."***

Response: We have revised the manuscript to address language inconsistencies and improve the narrative structure. Contextualization of findings has been enhanced in the Introduction, Interpretation, and Barriers to Attractiveness and Retention sections of the final manuscript.

- ***". Furthermore, as a minor point and as the authors acknowledge, data was collected in 2021 and 3 years later the findings, and there was a risk of non-trivial sampling bias, leaving these findings with some fragility.."***

Response: While we acknowledge the importance of updated data, the evaluation of attractiveness measures often requires a decade to fully assess their impact. As such, the conclusions drawn from the 2021 dataset remain relevant and reflective of current challenges. Future studies are planned to include more recent data and enable temporal analysis.

- ***In terms of writing, there are plenty of minor writing errors, which are easily correctable (e.g. incorrect use of tense), but there are sufficient number of significant writing deficiencies that leave me without confidence that they can be remedied. In totality, I think sufficient work is required here that the manuscript is not ready for publication. My recommendations are twofold. Firstly, I recommend repeating the survey; doing this would provide more data, and more contemporary data as well as the opportunity to conduct a temporal analysis. Secondly, I recommend employing strategies to really tighten up the writing.***

Response: We would like to thank reviewer 1 for this comment. We hope that this new version will provide the necessary insights for reviewers and readers of the journal to benefit from our work.

## 1.2. Specific Comments:

- ***Lines 15-17: "*** ***Not sure of the logic here. I agree that physiotherapists play a critical role in addressing the impacts of population aging, especially in hospitalized patients. The implied connection made here about the context of workforce shortages though is not clear. Are physiotherapists not vital, independent of workforce shortages? If workforce shortages didn't exist would the role of physiotherapists still not be vital? This just needs a little work in terms of logic progression.."***

Response: Thank you for your insightful comment. In accordance with your recommendation, we have rewritten the paragraph to clarify the logical progression leading to the issue of workforce shortages. The revised version ensures that the vital role of physiotherapists in addressing the impacts of population aging is highlighted independently of the workforce shortage context, while also logically connecting this role to the broader challenges posed by such shortages. We hope this revision better aligns with your expectations.

- ***Line 21: "*** ***Define FDHRS at its first use for the reader not familiar with the French context.."***

Response: Thank you. In accordance with the other comments, the sentence has been rewritten. The source "FDHRS" is now only referenced in the bibliography. Please see line 15 to 46 the corrected Introduction : *“This is driven by an aging population requiring more complex care, rising healthcare costs, and an insufficient number of qualified physiotherapists in public sector positions (7–9). These factors exacerbate the strain on public healthcare institutions and underscore the urgent need to address workforce challenges.*

*The attractiveness and retention of physiotherapists in the public sector have become critical issues in France (2,5). Several factors contribute to these challenges. One major issue is the wage disparity between public and private practice physiotherapists. In 2022, physiotherapists in private practice earned an average of €44,243, 30–50% (10) more than their counterparts employed in the public sector, who earned €32,808 on average (11). This wage gap creates a strong incentive for physiotherapists to choose private practice over public sector employment (12). In addition to financial considerations, public sector physiotherapists often face professional dynamics, management practices, and limited career advancement opportunities, all of which can further deter potential recruits and contribute to high turnover rates (5,13–15).*

*Moreover, the aging workforce, combined with the increasing number of retirements among experienced physiotherapists, exacerbates the shortage in the public sector (16,17). These factors, alongside the demanding nature of the work and the high patient load in public hospitals, make attractiveness even more challenging.*

*Between 2016 and 2021, ministerial measures were introduced to enhance salary scales and implement commitment bonuses for public sector employees. However, the effectiveness of these initiatives in addressing attractiveness and retention issues has not been formally assessed (18,19). Despite these efforts, attractiveness and retention challenges persist. The limited success of these measures suggests that a more comprehensive and sustained approach is needed (20,21). Policies have often focused more on nursing staff, with less emphasis placed on physiotherapists, which may have contributed to the ongoing difficulties in the physiotherapy workforce (19). Since 2015, French physiotherapists have been trained over five years, earning a Master’s degree. This reform has led to a substantial increase in their competencies and responsibilities. However, these advancements in qualifications and professional scope have not been matched by corresponding changes in their roles, responsibilities, or compensation within the public sector (22–24). »*

- ***Lines 23-25: "I am not convinced of the value of this statement. The data doesn't allow for firm interpretation (as the authors acknowledge), particularly given how the (health workforce) world has changed since 2020. Suggest removing."***

Response: as request, this section has been removed for clarity. Please see line 15 to 46 the corrected Introduction.

- ***Line 40-42: “Statement does not make sense. Please review/edit/remove. The narrative works (is better) with the statement simply removed.”***

Response: thank you. We have removed it.

- ***Line 47: “Reference for the 30-50% wage gap?”***

Response: The references have been added (lines 22-26).

Please see the new references below:

- Les salaires dans la fonction publique hospitalière en 2022 - Insee Première - 2015 [Internet]. [cited 2024 Dec 22]. Available from: https://www.insee.fr/fr/statistiques/8253303
- UNASA. Donnees statistiques sur la profession liberale en France [Internet]. 2021 [cited 2024 Dec 22]. Available from: https://www.unasa.fr/wp-content/uploads/2022/07/2021-Recueil-UNASA.pdf
- Flodgren G, Eccles MP, Shepperd S, Scott A, Parmelli E, Beyer FR. An overview of reviews evaluating the effectiveness of financial incentives in changing healthcare professional behaviours and patient outcomes - Flodgren, G - 2011 | Cochrane Library. [cited 2024 Dec 22]; Available from: https://www.cochranelibrary.com/cdsr/doi/10.1002/14651858.CD009255/abstract
- ***Lines 48-53: The wording is too strong here. Some suggested improvements: "At the institutional level, there is an opportunity need to develop localized strategies to improve fidelity and attractiveness and to tackle these issues effectively. Given the recruitment challenges in the profession, institutions might consider implementing approaches that emphasize project-based solutions and create a supportive environment for students."***

Response: Thank you for this suggestion, which we have added to the heavily modified introduction text.

- ***Lines 39-66: "*** ***This part of the narrative is confusing, I think due to lack of coherent structure. Paragraphs move backwards and forwards between describing the problem, then suggesting solutions, then returning to the problem again. This makes it hard for the reader to understand. Can I suggest you have a paragraph (or 2) focusing on the mechanisms that explain the recruitment and retention issues. Then, describe the historical strategies that have been employed (and their impact). This should connect the reader more seamlessly to the paragraph starting Line 67.."***

Response: In response to your comment, we have restructured the section to enhance coherence and improve the narrative flow. Following your suggestion, we have dedicated one paragraph to discussing the mechanisms underlying recruitment and retention issues, followed by a separate paragraph outlining the historical strategies employed and their impacts. These changes aim to create a more seamless transition to the paragraph. Please see line 15 to 46 the corrected Introduction.

- ***Lines 71-72: ""How can we implement measures..." This does not make sense. Do you mean strategies? You measure outcomes; you implements strategies (or initiatives, etc.). Please edit wording to better match intended meaning..'"***

Response: The wording has been revised for clarity. The terms "measures" and "strategies" have been used in the manuscript with specific intent: "measures" refers to specific actions or policies, while "strategies" denotes broader, overarching approaches. This distinction has been maintained throughout for clarity.

- ***Lines 75-76: You cite COVID as a contextual factor that may impact attractiveness of employed physiotherapy without referring to it in the background. If it is relevant it should have been introduced prior to here.***

Response: thank you for your feedback. covid does not seem relevant. we have removed this referring.

- ***Line 78: "*** The aim of this study was...***.'"***

Response: Corrected for grammatical accuracy. (See Introduction, line 47.)

- ***COVID Context (Lines 75-76):***"Introduce COVID earlier or remove."

Response: References to COVID-19 have been removed to maintain focus on the primary objective.

- ***Line 79: "... that may encourage..."***

Response: it is corrected.

- ***Lines 80-82: The description of the outcome measure is not necessary or helpful here. Relocate to methods. Edit lines 82-84 for the same reasons as some content here relates more to methods.***

Response: it’s done.

- ***Line 82- "The study examined..."***

Response: it’s corrected.

- ***Line 85: "we sought to..."***

Response: it’s corrected.

- ***Line 89: Materials and Methods***

Response: it’s corrected.

- ***Line 117: "They had..."***

Response: it’s corrected.

- ***Materials and Methods: "*** ***I would like to see the entire questionnaire as part of the main manuscript. Reference is made to certain sections in the 'Results', but I think it important to include for reference. Please include and as part of the manuscript rather than as an appendix.***

Response: A table summarizing survey questions has been added (please see line 107 to 108 in Materials and Methods session, Table I). The table is presented below.

**Table I: Questionnaire of the survey**

***PART I: General demographic questions***

***PART II: Feelings and Experiences in Daily Work***

*Question: "Do you agree with the following statement?"*

*Response options: "Strongly agree," "Agree," "Neutral," "Disagree," "Strongly disagree”.*

1. My institution faces an issue with the attractiveness of physiotherapist positions.

2. The attractiveness measures implemented by successive governments since 2016 have been sufficient.

3. You experience difficulties or feel overwhelmed in your daily work.

***PART III: Opinion About Attractiveness Measures***

*Question: "Do you agree with the following statement?"*

*Response options: "Strongly agree," "Agree," "Neutral," "Disagree," "Strongly disagree."*

**Domain 1: Adoption of National Measures**

1. Wage increases enhance employee attractiveness.

2. Recognizing a Master's degree for physiotherapy studies enhances employee attractiveness.

3. Recognizing additional diplomas in the public hospital service enhances employee attractiveness.

**Domain 2: Inclusion in Daily Structure**

4. Including physiotherapists in the development of rehabilitation projects enhances employee attractiveness.

5. Including physiotherapists in shared inter-facility projects enhances employee attractiveness.

6. Including physiotherapists in the management of their facility enhances employee attractiveness.

**Domain 3: Supervision of Employed Physiotherapists**

7. Having a healthcare manager from one of the rehabilitation professions enhances employee attractiveness.

8. Having a senior healthcare executive from one of the rehabilitation professions enhances employee attractiveness.

9. Entrusting cross-functional activities to physiotherapists (e.g., ethics, steering commissions, working groups) enhances employee attractiveness.

**Domain 4: Quality of Life and Mentorship**

10. Involving physiotherapists in tutoring trainees enhances employee attractiveness.

11. The quality of work life is an important factor in choosing your professional activity.

12. Management consideration of personal quality of life promotes employee attractiveness.

- ***Figures and Tables: "*** ***Table III and Figure 3 present the same data in slightly different ways. I see no reason to have both, the Table is easier to interpret, and the Table is referred to in the main text more than the Figure. Remove either, and I suggest removing Figure 3.."***

Response: Figure 3 has been removed. Please see Results, line 165.

- ***Line 213: Poor title name***

Response: we have corrected it by the following title: “Effect of age and current profession on socio-professional outcomes (n=448)”

- ***Lines 224-228 (or even 233): This is a repetition that I don't think is necessary. Suggest removing of trimming significantly.***

Response: it’s deleted.

- ***Lines 234-241: "*** ***This paragraph doesn't make sense. You don't frame the paragraph with an introductory sentence, nor any concluding statement. You make various disparate connections between findings and phenomena. Many of which don't make sense. For example, you make a connection between the 'issue of attractiveness' and findings related to low percentage 'feeling overwhelmed'. I simply don't understand this connection. You go on to cite this as a risk factor for of 'psychosocial issues' and then quality of work-life. I appreciate that theoretically there are relationships between many of these phenomena, but you do not make this clear and you do not align theory with your data in a way that might enable the reader to conclude how your data has influenced theory. This paragraph needs substantial re-working.."***

Response: The paragraph has been rewritten for clarity and coherence. It now begins with an introductory sentence explaining the connection between the "issue of attractiveness" and psychosocial risks. The relationship between feeling overwhelmed and quality of work-life has been better aligned with the data and contextualized within relevant theories. A concluding sentence ties these findings to the broader discussion on workforce challenges. Please see the corrected Discussion line 234 to 240:

*“The study revealed several barriers that reduce the attractiveness of employed physiotherapy positions. Inadequate compensation and lack of recognition for advanced qualifications emerged as critical issues. Respondents under 40 years old were particularly sensitive to the lack of career development opportunities and the limited integration of physiotherapists into institutional decision-making. These findings align with prior literature emphasizing the importance of aligning professional roles with advanced training and qualifications (35–37).”*

- ***Lines 249-252: "."...not yet seized the opportunity..." this is political speculation/judgement. I don't disagree with your sentiment but you shouldn't deploy statements such as this or in this way; author bias rings through in its current form. You go on to speculate regarding the impact of 'this failure'. This may be true (or not) but is currently speculative and overstated in the context of your research question and data. This speculation needs tempering”..***

Response: This section has been revised to temper the speculative tone. The phrasing has been adjusted to reflect a neutral and evidence-based interpretation of the findings, avoiding language that may imply bias or overstatement. The revised paragraph now emphasizes observed gaps and their potential implications without attributing failure or assigning blame. Please see the corrected Discussion line 252 to line 253: « Based on the survey findings and literature (41,42), the study proposed ten actionable measures to enhance the attractiveness of employed physiotherapy positions (Table VI).”

- ***Lines 264-269: "Once again this paragraph touches briefly on some big issues but without thoughtful explanations and connection to the research question or data. For example, to state 'evidence-based practice' as a change in the profession is simply too broad and vague to be useful. This paragraph needs work."***

Response: The paragraph has been expanded and clarified to better connect the discussion of "evidence-based practice" and professional changes to the research data. Specific examples have been included to ground the discussion in observed trends, and the implications of these changes are now explicitly linked to the study findings. Please see the corrected Discussion line 264 to line 275: “This study highlights the pressing need for systemic changes to improve the attractiveness and retention of physiotherapists in French public hospitals. Key recommendations include salary adjustments, professional recognition, and policies supporting work-life balance and mentorship. These measures address barriers such as inadequate compensation and limited career opportunities, aiming to align professional roles with evolving competencies and workforce expectations.

The challenges identified are not unique to France; literature shows that similar issues are prevalent in other countries. The evolving role of physiotherapists and their enhanced qualifications make this an increasingly relevant global concern. Addressing these challenges is essential to ensuring a sustainable workforce and high-quality care in healthcare systems worldwide.“

# 2. Reviewer #2

## 2.1. General Comments:

- ***Thank you for the opportunity to review this manuscript ‘Attractiveness and Retention Factors for Employed Physiotherapists in France: A Multicentre Survey’. This observational cross-sectional survey study provides insights into physiotherapists’ perspectives on the attractiveness of working in public health. The authors are to be commended for exploring this important area and achieving a great response to their survey, however some changes are needed to enhance the clarity of this work and its findings.***
- ***"*** ***I have interpreted the term ‘employed physiotherapy’ to mean working as a physiotherapist within the public health sector in France. This terminology requires defining or rephrasing for the manuscript, as the term alone may not convey the same meaning internationally.."***

Response: The terminology has been revised to "physiotherapist employed in the public sector," with a clear definition in the introduction. (See Introduction, lines 20-29).

- ***"In some sections, the terms ‘attractiveness’ and ‘retention’ are used inconsistently and could be refined. These terms are not always interchangeable, leading to potential confusion. For instance, the abstract primarily mentions ‘attractiveness,’ as does most of the manuscript (e.g., Lines 130-132, which state the domains aimed at ‘improving the attractiveness of employed physiotherapy’). However, the title references ‘retention’ as well. Additionally, Line 99 mentions that the inclusion criteria were designed to ‘gain insights into retention influencing factors.’ Maintaining a consistent focus on ‘attractiveness’ throughout would enhance readability.'"***

Response: These terms have been defined and used consistently. (See Introduction and Discussion.)

## 2.2. Specific Comments:

- ***Introduction “The authors might consider restructuring the introduction. Could the second paragraph serve as a stronger opening for the manuscript?"***

Response: In response to your comment, we have restructured the section to enhance coherence and improve the narrative flow. Following your suggestion, we have dedicated one paragraph to discussing the mechanisms underlying recruitment and retention issues, followed by a separate paragraph outlining the historical strategies employed and their impacts. These changes aim to create a more seamless transition to the paragraph beginning on Line 67, and we hope this revision addresses your concern. (See Introduction, lines 1-46.)

- ***Lines 39-42: "*** ***The statement that government strategies have been in place since 2015 requires more detail. This is mentioned again in the discussion, and providing more context would help readers understand whether your final recommendations align with or differ from previous efforts."***

Response: Additional details on ministerial measures, including salary scales and bonuses, have been included. (See Introduction, lines 34-37.)

- ***Geographical Challenges: "*** ***Are there any geographical challenges to public health employment in France? This aspect is not specifically addressed and could be important to mention "***

Response: This aspect was not investigated but is acknowledged as an area for future research.

- ***The introduction should focus on setting up the background and research question. The explanation of the primary and secondary outcome measures (lines 80-84) is not needed, as it appears (appropriately) in the methods section. (In addition, it does not need repeating in the discussion).***

Response:

- ***Lines 95-97: The statement regarding the minimum number of responses needs further clarification. This is exploratory observational research conducted via a cross-sectional survey, and no hypotheses are being statistically tested. This is a large sample for survey results, and it can be explored in other ways (e.g. via its characteristics) to argue it is a representative sample of the relevant population.."***

Response: The sample size calculation and its representativeness are clarified. (See Materials and Methods, lines 76-81.)

- ***I would appreciate more details on the survey development process, particularly how and why the four domains were chosen. Including a copy of the entire survey, with statements related to each domain, would be helpful.***

Response: Thank you for your question. To answer the first part, the four domains were chosen with the intent to link national strategies to the realities faced by physiotherapists in their day-to-day work, moving from a macro (national) perspective to a micro (local) one. The aim was to explore whether, beyond government measures, local factors and workplace dynamics could influence the attractiveness and retention of employed physiotherapists. The first domain focuses on the adoption of national measures and how these policies are being implemented within the profession. The second domain looks at the inclusion of physiotherapists in the daily operations of their organizations, assessing how well they are integrated into their teams and the impact this has on their job satisfaction. The third domain examines the role of supervision and mentorship for physiotherapists in the workplace, acknowledging the importance of strong leadership in supporting their professional development. Finally, the fourth domain considers the overall quality of life and the role of mentoring in the retention of physiotherapists, addressing factors such as work-life balance and support systems that contribute to long-term career satisfaction. By focusing on these domains, the survey aimed to investigate whether local conditions, independent of national policies, can play a role in improving the attractiveness and retention of physiotherapists in the workforce.. To explain this, it was added in the text (lines 104-109): *"This four domains were chosen to provide a holistic view of the factors influencing the attractiveness retention of physiotherapists in the public sector. By exploring both national-level policies and the daily realities of physiotherapists in hospitals, we aimed to identify opportunities for improvement at every level. The survey thus sought to determine whether local practices could independently influence retention and attractiveness, independent of broader government measures. "*

In order to clarify the second part of your question, you will find the table 1 of completed questionnaires. We hope that this answer will help you with your request.

- ***"*** ***I would appreciate more details on the survey development process, particularly how and why the four domains were chosen. Including a copy of the entire survey, with statements related to each domain, would be helpful.”***

Response: Additional details have been added to describe the rationale behind the selection of the four domains, which were informed by a review of the literature and expert consultations.

- ***Lines 117-118: “***Please clarify how data could be removed upon a participant’s request if it was anonymous. Was the data anonymised after collection, or were all responses anonymous from the start, as suggested in Lines 109-110?***”***

Response: Participants were also informed that they could withdraw their consent at any time up to the end of the data collection process. Validation renders the data anonymous, making it impossible to erase it afterwards (lines 124-126).

- ***Lines 141-142: Please revise the term ‘qualitative variable’ as the statements collect Likert scale (categorical) data, not qualitative responses.***

Response: thank you for your comment. we've changed our inaccuracy.

- ***Lines 146-149: Please clarify how the secondary outcomes were examined. The results mention Chi-square analysis, which is suitable for categorical data. What categories were used for ‘age’? Adding a rationale for this analysis would help clarify its connection to your research question.***

Response: Response: (Line 116) For secondary outcomes, a chi-square test was conducted to assess the relationship between demographic factors, such as age and profession, and responses to the survey questions. (lines 186-189) : Statistical analysis revealed significant relationships between age and certain variables, particularly for participants under 40 years old, who placed greater importance on the recognition of additional qualifications, work-life balance in career choices, and quality of life considerations in assessing job attractiveness.

## 2.3. Results

- ***Lines 155-176: The inclusion criteria specify ‘physiotherapists working in public health,’ yet, some managers identified as nurses? Is this correct?***

Response: Managers from other professions were excluded to maintain focus on physiotherapists. (See Materials and Methods, lines 70-73.)

- ***Please ensure that the units of measurement are clearly indicated in all tables, either in the title or the column headings.***

Response: we have thoroughly reviewed and completed all the tables, ensuring that the units of measurement are clearly indicated either in the title or in the column headings.

- ***Does Figure 3 duplicate the information in Table III? Are both necessary? Additionally, the use and interpretation of confidence intervals for Likert scale data need clarification.***

Response: Figure 3 has been removed. Please see Results, line 165.

- ***Lines 196-200: Besides clarifying the age categories, this section could elaborate on the findings. For instance, which age categories showed significant re : “lationships? Was an older age associated with higher Likert scale ratings on these items?***

Response: The rationale and methods for analyzing these variables have been expanded. (See Materials and Methods, line 141; and results line 190.): ”Statistical analysis revealed significant relationships between age and certain variables, particularly for participants under 40 years old, who placed greater importance on the recognition of additional qualifications, work-life balance in career choices, and quality of life considerations in assessing job attractiveness.”.

- ***The titles of items 24 and 25 differ between Table IV and lines 196-199. Please ensure consistency.***

Response: thank you for your comment. For clarity and to avoid confusion, the question numbers have been removed and replaced with the thematic name of the question.

## 2.4. Discussion

- ***This section would benefit from revision. The first two paragraphs summarise the introduction and methods, but the authors could just start with directly summarising their main findings - then expanding on pertinent points. Lines 234-241: The authors could clarify how the low proportion of respondents who never felt overwhelmed relates to the issue of attractiveness. Additionally, it is unclear from the results where teamwork, interdisciplinary work, and a sense of belonging were significant. A stronger link to the background and results is needed.***

Response: These sections have been expanded and directly linked to findings. (See Discussion, Barriers to Attractiveness and Retention, lines 234-240.)

- ***More detail is needed on how the measures in Table V were derived, including how the study results informed them. For example, the results (Lines 192-195) indicate that mentoring involvement was less significant than work-life balance and professional quality of life, yet measures 7 and 8 focus on supervision and training related to mentoring. More information is needed on what has been previously implemented (e.g. since 2015) and how the suggested measures compare.***

Response: (summarized lines 256-257). The justification for the ten recommended measures to enhance the attractiveness of employed physiotherapy positions is supported by specific findings from the survey data, which highlight key issues and areas of improvement. Here are the justifications with corresponding data:

1. Salary Adjustments:

Justification: 48.9% of respondents strongly agreed, and 41.1% agreed that wage increases would enhance employee attractiveness (Table IV). This strong agreement indicates that improving compensation is a primary factor for improving the appeal of physiotherapy positions, especially in the context of the study's finding that 64.1% disagreed with the sufficiency of government measures to address salary issues since 2016.

2. Recognition of Additional Qualifications:

Justification: 62.3% of participants indicated that recognizing additional qualifications, such as Master’s or specialized diplomas, would significantly enhance attractiveness (National Measures). Furthermore, 21.9% strongly agreed and 26.3% agreed that recognizing a Master's degree for physiotherapy studies would improve attractiveness (Table IV). This shows the importance of aligning professional recognition with academic advancements.

3. Development of Institutional Policies Supporting Rehabilitation Professions:

Justification: 79.9% of respondents agreed or strongly agreed that their institution faces issues with attractiveness, highlighting the need for institutional changes to improve this. In addition, including physiotherapists in rehabilitation project development was supported by 90.6% of respondents as a way to enhance attractiveness (National Measures). This demonstrates the positive impact of more inclusive and supportive institutional policies.

4. Fostering Interdisciplinary Collaboration:

Justification: 68.3% of respondents believed that involving physiotherapists in shared inter-facility initiatives would enhance attractiveness (National Measures). Additionally, 50.2% strongly agreed and 40.4% agreed that physiotherapists should be included in rehabilitation project development, indicating the importance of cross-functional collaboration in improving job satisfaction.

5. Increased Management Involvement:

Justification: 73.6% of respondents agreed or strongly agreed that having a clinical supervisor or senior healthcare executive with rehabilitation training would improve attractiveness (National Measures). This data supports the recommendation to increase managerial involvement, particularly by placing rehabilitation professionals in key leadership roles.

6. Promotion of Work-Life Balance:

Justification: 80.8% of respondents agreed that the quality of work-life is an important factor in choosing their professional activity (Table IV). This is reinforced by the finding that 94.6% of participants agreed that professional quality of life is a critical factor for job attractiveness (Individual Factors), showing that promoting work-life balance is essential for retention.

7. Mentorship Programs:

Justification: 61.4% of respondents perceived mentorship as an important factor for job satisfaction (Individual Factors). Although this is less emphasized than other factors, it still represents a significant portion of the respondents who see value in mentorship and professional guidance, which can improve retention rates.

8. Career Development Opportunities:

Justification: The study found that respondents under 40 years old were particularly sensitive to the lack of career development opportunities (Table V). In particular, 49.3% of participants reported feeling "often" or "always" overwhelmed in their daily work, likely indicating a need for more structured career development and growth opportunities to reduce burnout and enhance job satisfaction.

9. Involvement in Rehabilitation Project Development:

Justification: 90.6% of respondents agreed that involving physiotherapists in rehabilitation projects enhances job attractiveness (National Measures). Additionally, 50.2% of respondents strongly agreed and 40.4% agreed that being included in rehabilitation projects increases job satisfaction (Table IV), confirming the importance of giving physiotherapists a central role in the development of these initiatives.

10. Institutional Support for Personal Quality of Life:

Justification: 63.8% of respondents agreed that management consideration of personal quality of life promotes employee attractiveness (Table IV). This reinforces the need for institutions to support employees’ personal well-being, as a majority of respondents felt that management's attention to their quality of life would significantly influence their job satisfaction and retention.

- ***The upgrade of entry-level education to a Master’s degree is mentioned in the introduction (Lines 57-58) and the discussion (Lines 246-247). Not needed as repetition in the discussion.***

Response: We have corrected it Please see the discussion line 223 to 224: *“Despite the implementation of a Master’s degree for physiotherapists in France since 2015 (15), public institutions have yet to fully integrate this elevated status into their operational and managerial frameworks.”*

- ***A limitation of the study is the use of an unvalidated survey.***

Response : We have added in the limitation part the following sentence line 210 to 211: “This study has several limitations that should be considered when interpreting the results. First, the survey instrument used was not formally validated, which may introduce biases in the data collection process.”

- ***Also, were participants asked to suggest additional measures or areas influencing attractiveness? (i.e. that weren’t already in the survey questions?) this is another limitation.***

Response : We have added the following paragraph line to line234 to 240 : “The study revealed several barriers that reduce the attractiveness of employed physiotherapy positions. Inadequate compensation and lack of recognition for advanced qualifications emerged as critical issues. Respondents under 40 years old were particularly sensitive to the lack of career development opportunities and the limited integration of physiotherapists into institutional decision-making. These findings align with prior literature emphasizing the importance of aligning professional roles with advanced training and qualifications (35–37).”

- ***A stronger conclusion that references the research questions and main findings may be needed.***

Response: The conclusion paragraph has been expanded and clarified to better connect the discussion of "evidence-based practice" and professional changes to the research data. Specific examples have been included to ground the discussion in observed trends, and the implications of these changes are now explicitly linked to the study findings. Please see the corrected Discussion line 264 to line 275: “This study highlights the pressing need for systemic changes to improve the attractiveness and retention of physiotherapists in French public hospitals. Key recommendations include salary adjustments, professional recognition, and policies supporting work-life balance and mentorship. These measures address barriers such as inadequate compensation and limited career opportunities, aiming to align professional roles with evolving competencies and workforce expectations.

The challenges identified are not unique to France; literature shows that similar issues are prevalent in other countries. The evolving role of physiotherapists and their enhanced qualifications make this an increasingly relevant global concern. Addressing these challenges is essential to ensuring a sustainable workforce and high-quality care in healthcare systems worldwide.“

- ***Recommendations: "Align recommendations with data."***

Response: Recommendations have been directly linked to survey findings. (See Recommendations, lines 252-260 ; Table VI.)

We would like to thank the reviewers for their comments, which have greatly improved our work and its understanding. We hope that this new version will fully meet your expectations.
